# Allosteric inhibition induces an open WPD-loop: a new avenue towards glioblastoma therapy†

**DOI:** 10.1039/c8ra08427k

**Published:** 2018-11-30

**Authors:** Clement Agoni, Pritika Ramharack, Mahmoud E. S. Soliman

**Affiliations:** Molecular Bio-computation and Drug Design Laboratory, School of Health Sciences, University of KwaZulu-Natal Westville Campus Durban 4001 South Africa soliman@ukzn.ac.za +27 (0) 31 260 7872 +27 (0) 31 260 8048

## Abstract

The mobility of loops around the catalytic site of a protein remains crucial to its activity. Dynamics of the WPD-loop is an essential determinant of the catalytic activity of tyrosine-protein phosphatase zeta, an implicated protein in glioblastoma cells. The WPD-loop assumes a closed conformation upon substrate binding in order to position its catalytic aspartate to participate in catalysis. Herein, we explore the impact of NAZ2329, a recently identified allosteric inhibitor of tyrosine-protein phosphatase zeta, on the atomic flexibility of the WPD-loop. The druglikeness of NAZ2329 was assessed using the SwissADME online tool. The enzymatic complex was then subjected to conformational simulations using the AMBER molecular dynamics software. Structural analysis revealed that NAZ2329 induced an open conformation of the crucial WPD-loop, consequently impeding enzyme activity even upon substrate binding. Based on the molecular interactions between NAZ2329 and tyrosine-protein phosphatase zeta, a pharmacophore model was generated to exhibit the important functional moieties of NAZ2329. These findings provide an insightful molecular and structural mechanism in targeting tyrosine-protein phosphatase zeta as a therapeutic intervention for glioblastoma. We believe that this optimized pharmacophoric model will aid in the design of improved anti-tyrosine phosphatase agents, thus allowing for increased patient adherence.

## Abstract 2

Structural mechanism of inhibition of NAZ2329 at the allosteric site of PTPRZ, with particular emphasis on the dynamics of the WPD-loop.

## Graphical abstract

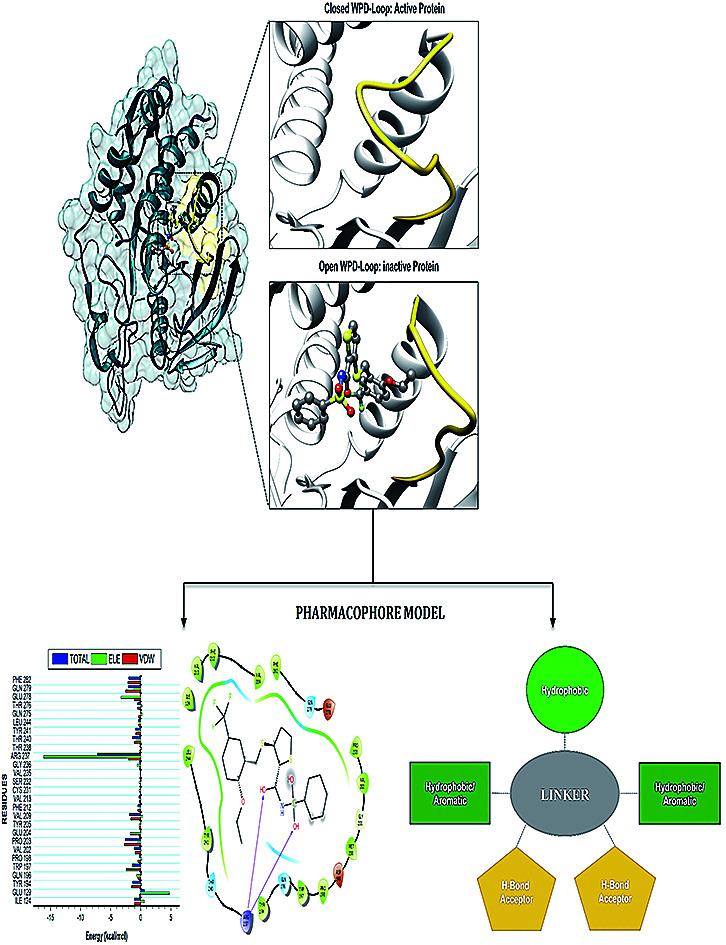

## 1 Introduction

Glioblastoma is classified as the most aggressive and frequently diagnosed central nervous system malignancy, with an annual incidence of 100 000 new cases, globally.^1^ Adding to the complexity of the disease, glioblastoma is associated with poor prognosis and low survival rates. Glioblastomas are tumors that arise from astrocytes in the cerebral cortex, but may be found anywhere in the brain or spinal cord.^2^ The tumors are highly vascularized to allow oxygen and nutrients to pass through, thus increasing their growth.^3^ Currently, there are no preventative therapies or cure for the disease, with only surgical resection and adjuvant chemotherapy being available.^4^

**Fig. 1:**
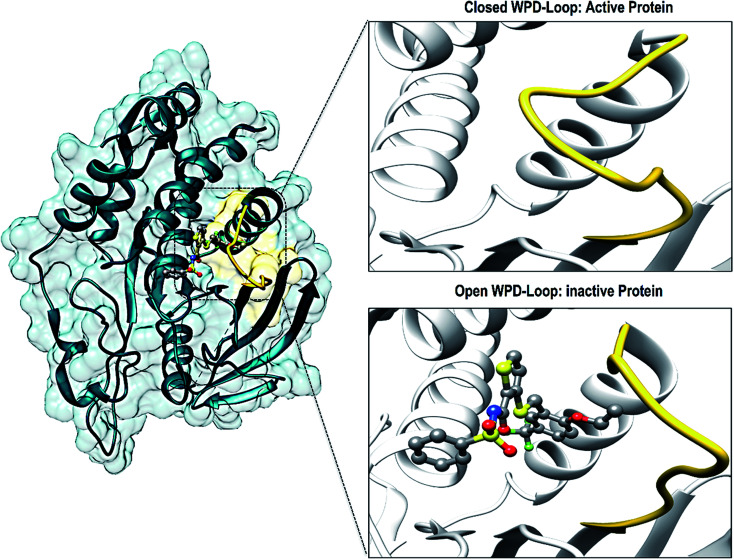
Graphical representation of the WPD-loop (yellow) of PTPRZ. The active form of the protein is associated with a closed WPD-loop upon binding of the natural substrate, whereas, an open conformation of results in an inactive protein.

There have been numerous studies elucidating the development of new therapeutic approaches, including an article that investigated the receptor type tyrosine-protein phosphatase zeta (PTPRZ) as a target in glioblastoma cells.^5^ The PTPRZ enzyme plays a crucial role in regulating protein tyrosine phosphorylation, thereby leading to the survival of the glioblastoma cells and promoting the growth of tumors.^6^ Due to this mechanism of action, the PTPRZ enzyme is a crucial target in the design of efficient inhibitors of against glioblastoma.^3^ Structurally, active site architecture of PTPRZ is made up the phosphate binding P-loop, the catalytic acid/base aspartate containing WPD-loop, the conserved glutamine containing Q-loop, the pTyr-recognition pTyr-loop and the multiple conserved residues containing E-loop.^7^ As a vital component of the catalytic process of PTPRZ, the WPD-loop serves as a flexible gate to catalytic site which is shown to assume a closed conformation in active protein form and an open conformation in inactive protein form (Fig. 1).^8^

Upon binding of a substrate to the active site the, the WPD-loop assumes a close conformation to position its catalytic aspartate to participate in catalysis.^7^ Therefore a compromised WPD-loop mobility can substantially decrease the catalytic activity of the overall PTPRZ protein as was reported by studies with mutated tryptophan hinge residue.^9,10^

There have been multiple phosphotyrosine competitive inhibitors that have been designed to inhibit the PTPRZ enzyme. These compounds, however, have failed to successfully attenuate cancer due to the drugs inability to permeate the cell wall.^11^ Another challenge when designing effective inhibitors of the enzyme is its highly conserved, positively charged active site. Due to these reasons, the enzyme was long known as an “undruggable target”.^5^

Over recent years, targeting the allosteric site of the PTP family of enzymes has proven to be successful.^12^ In 2017, a cell-permeable small molecule, NAZ2329, was identified to allosterically inhibit both the PTPRZ and PTPRG, thus mitigating the tumorigenicity in glioblastoma cells.^13^ This allosteric inhibition strategy has since then shown to be extremely promising in glioblastoma drug design.

Here we assess, through the use of *in silico* tools, the structural mechanism of inhibition of NAZ2329 at the allosteric site of PTPRZ, with particular emphasis on the dynamics of the WPD-loop. Also, a pharmacophore model was design based on the binding profile and structure activity relationship of NAZ2329 with PTPRZ. This pharmacophore model approach will facilitate the design of small molecule inhibitors that will not only target PTPRZ and PTPRG, but will be applicable to other tyrosine phosphatases as well.

## 2 Computational methods

### 2.1 Exploring the drug likeliness of NAZ2329

SwissADME,^14^ an online software, was used to assess the physicochemical descriptors, the pharmacokinetic features and the drug-worthy nature of NAZ2329. In computing the lipophilicity and polarity of NAZ2329, the “Brain Or Intestinal Estimated permeation, (BOILED-Egg)” method was employed.^14^

### 2.2 Preparation of PTPRZ system

To identify the most suitable crystal structure for molecular dynamic simulations, all PTPRZ structures available on RSCB Protein Data Bank^15^ were assessed. Of the eight structures, only four were of human origin. As our study focused on the NAZ2329 molecule bound to the allosteric site of PTPRZ, the PDB code 5AWX was chosen as the most suitable crystal structure. Based on the literature accompanying the crystal structure, it was noted that the free enzyme demonstrated a closed WPD-loop, whilst an open WPD-loop was depicted in the NAZ2329-bound complex. This justified the use of both the bound crystal structure as well as the free enzyme (PDB codes: 5AWX and 5H08, respectively).^16^

To validate the binding site of PTPRZ, the NAZ2329 molecule was extracted from the complex using the UCSF Chimera software package.^17^ Molecular docking was then carried out using the Autodock Vina plugin on the Chimera software, where default settings were used. The grid box was defined around the following key residues Glu1981, Arg1939, Gly1938, Pro1905, Glu1898, Trp1899, Val1911 and Glu1977, which covered the allosteric region of PTPRZ. The *X*, *Y* and *Z* centre values of the grid box were defined as 30.58, −2.11 and 43.21, respectively, whereas, the *X*, *Y* and *Z* size dimensions were defined as 10.8, 14.3 and 11.4, respectively. Docking results, of binding affinity −9.7 kcal mol^−1^, indicated significantly similar binding poses of the original crystal structure to the docked NAZ2329 (Fig. S1†). This validated the docking pose, thus allowing further analysis to be carried out. The structures of PTPRZ and NAZ2329 were then prepared and missing residues modeled using the UCSF Chimera software package.^17^

### 2.3 Molecular dynamic (MD) simulations

Molecular dynamic (MD) simulations present a vigorous tool to explore the physical movements of atoms and molecules, and in so doing, unveiling dynamical evolution of biological systems. Using the GPU version of the PMEMD engine provided with the AMBER package, MD simulation was carried out in which the FF14SB variant of the AMBER force field.^18^

Atomic charges for NAZ2329 were created by employing the Restrained Electrostatic Potential (RESP) and the General amber Force Field (GAFF) in ANTECHAMBER. Neutralization of all systems and addition of all hydrogen atoms was performed by using the Leap module incorporated in the AMBER 14 package. Na^+^ and Cl^−^ served as counter ions for the neutralization process. As per Leap's default settings, the amino acids were renumbered in a sequential order, so that residue “G1696” was renamed as “G1”. Results were thus displayed as per renamed residues. Using TIP3P water molecules of 8 Å box size, all systems were implicitly solvated.

Minimizations of the systems were carried out in two separate stages. The first stage involved a 2000 steps minimization with an incorporated restraint potential of 500 kcal mol^−1^ Å^−2^. The second stage involved a 1000 steps full minimization process incorporating a conjugate gradient with no restrain.

All systems were then steadily heating from 0 K to 300 K over 50 ps, in a manner that ensured that all systems such that the systems conserved a fixed atom number and volume. Solutes in the systems were given a potential harmonic restraint. A potential harmonic restraint of 10 kcal mol^−1^ with a collision frequency of 1.0 ps was imposed on solutes in all systems. Equilibrations of all systems were then performed after heating over a 500 ps period at a constant operating temperature of 300 K. Constant pressure and atom numbers were also ensured by mirroring an isobaric–isothermal ensemble (NPT). A 1 bar pressure was maintained for all systems using the Berendsen barostat.

A 100 ns MD simulation was carried out on all systems in which the SHAKE algorithm was incorporated to constrict bonds of hydrogen atoms. A 2 fs simulation step coupled with a SPFP precision model was used. The simulations coincided with isobaric–isothermal ensemble (NPT), with randomized seeding, constant pressure of 1 bar maintained by the Berendsen barostat, a pressure-coupling constant of 2 ps, a temperature of 300 K and Langevin thermostat with collision frequency of 1.0 ps.

### 2.4 Post-dynamic analysis

The PTRAJ module of AMBER14 suit was used to analyze all trajectories generated from coordinates at every 1 ps from all simulated systems. Also, the CPPTRAJ module of AMBER14 package was used to analysis of RMSD, RMSF and Radius of Gyration.

#### 2.4.1 Binding free energy calculations

The binding free energy generated in the simulated systems was estimated using the Molecular Mechanics/GB Surface Area approach as has been employed in some of our previous reports.^19–21^ The estimated binding free energies may expound the binding mechanism between NAZ2329 and PTPRZ. From the 100 ns simulation trajectory, 10 000 snapshots were obtained and subsequently used to estimate the binding free energy. By the MMGBSA approach, the binding free energy is estimated for the complex, ligand and receptor as follows;

1 Δ*G*_bind_ = *G*_complex_ − *G*_receptor_ − *G*_ligand_

2 Δ*G*_bind_ = *E*_gas_ + *G*_sol_ − *TS*

3 *E*_gas_ = *E*_int_ + *E*_vdw_ + *E*_ele_

4 *G*_sol_ = *G*_GB_ + *G*_SA_

5 *G*_SA_ = *γ*SASA

*E*_gas_ represents gas-phase energy and is made up of the sum total of the internal energy *E*_int_; Coulomb energy *E*_ele_ and the van der Waals energies *E*_vdw_. *G*_sol_ represents the solvation energy which is estimated by the sum total of polar state energy contributions, *G*_GB_ and non-polar energy contributions, *G*_SA_. Using a water probe radius of 1.4 Å, *G*_SA_ is estimated from the solvent accessible surface area (SASA). *G*_GB_ is determined from the GB equation. Total entropy of solute and temperature is represented as *S* and *T* respectively. The accuracy of the estimated relative binding free energies reported here might be enhanced if the terms in the eqn (2) are averaged over multiple conformations or MD snapshots,^22^ but this usually dependent on the research interest under consideration. Conducting MD simulations for NAZ2329, PTPRZ and the complex will yield more accurate results in the calculating the binding free energies; it requires greater computational resources, which were not readily available for this study.

#### 2.4.2 Dynamic cross-correlation analysis (DCC)

The correlation coefficient of motions between the atoms in PTPRZ over the simulation period were quantified by calculating the dynamic cross correlation matrix.^23^ DCC was performed using CPPTRAJ module of the AMBER 14 suite. The formula used to describe dynamic cross correlation is given below:
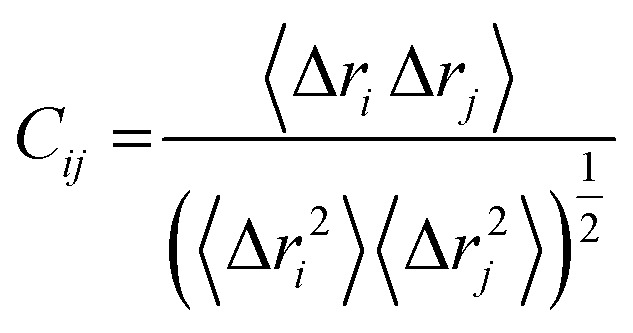
where, *i* and *j* represents the *i*^th^ and *j*^th^ residue respectively. The displacement vectors that correspond to the *i*^th^ and *j*^th^ residue respectively is represented as Δ*r*_*i*_ or Δ*r*_*j*_.The Origin software^24^ was then used to plot the DCC matrix.

#### 2.4.3 Principal component analysis (PCA)

Principal component analysis (PCA), is a covariance-matrix-based approach that is used to show the displacement of atoms and the dynamics of loops of a protein.^25^ Using the PTRAJ module of the AMBER14 package, solvent water molecules and neutralizing ions added by the Leap module are stripped prior to MD trajectory generation. There is then an alignment of the stripped trajectories against their respective fully minimized structures. PCA was performed for C-α atoms on 1000 snapshots each. The first two principal components and covariance matrices were calculated using scripts developed with our research group. PCA calculations were conducted over 1000 snapshots each for C-α atoms. PC1 and PC2, which represent the first two principal components, are created from the trajectories averaged from the unbound PTPRZ and the bound system. Using Cartesian coordinates of C-α atoms a 2 × 2 covariance matrix is created. The first two eigen vectors of covariant matrices correspond with the created PC1 and PC2. The Origin software^24^ was then employed to create PCA plots.

### 2.5 Pharmacophore model creation

Following the simulation of NAZ2329 at the active site of PTPRZ, per-residue energy decomposition analysis was used to determine the amino acids that contribute the most towards the binding of NAZ2329. In constructing the pharmacophore in this study, the pharmacophoric moieties that exhibited prominent interaction with highest energy contributing amino acids were used. In validating and generating our model, it was uploaded on the ZincPharmer^26^ and LigandScout.^27^

## 3 Results and discussion

### 3.1 Sequence analysis and structural stability of PTPRZ

**Fig. 2:**
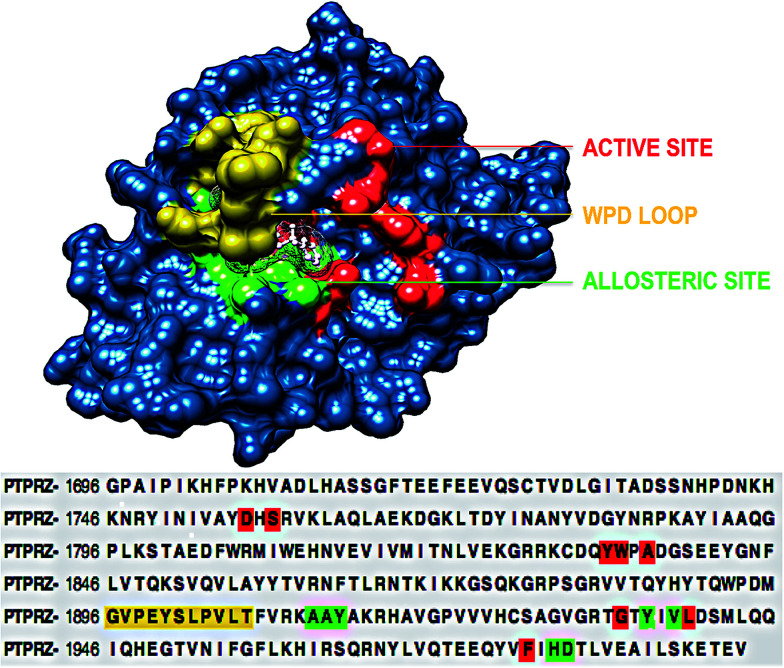
Structural and corresponding sequence representation of the unique regions of the PTPRZ protein; active site-red, allosteric site- green and WPD-loop-yellow (PDB code: 5H08).^16^

To understand the structural mechanism of inhibition of PTPRZ, it is important to identify the fundamental structural characteristics of the protein. Based on previous studies, it has been established that the active and allosteric sites may be found adjacent to each other. The WPD-loop, a vital catalytic site regulator, forms an outer cover to part of the allosteric site, indicating that the ligand that binds to this allosteric pocket would govern the dynamics of this loop. Fig. 2 graphically represents these unique structural features, including the corresponding amino acids for each region.

PTPRZ as a protein expressed in the central nervous system is localized in the glial cell where it reportedly mediates cell adhesion signaling events during neurogenesis. The strong expression of PTPRZ glioblastoma^28^ renders them as viable anti-cancer therapeutic targets treatment. The inhibitory activity of NAZ2329 as experimentally reported, could potentially influence the conformational stability of PTPRZ.^5^

As such, the stability of 3-D backbone atoms of the simulated APO and complexed PTPRZ was ascertained by calculating the root mean standard deviation (RMSD) of the generated trajectories over the 100 ns simulation period. Calculated RMSD also assessed the convergence of the respective systems as depicted in Fig. S1,† with each system assuming an energetic plateau after about 22 ns. An initial increase in RMSD to 3.4 Å in the APO system between 0 to 20 ns was observed, illustrating the dynamic conformational changes associated with the expansion of PTPRZ over that period. The expansion allowed infiltration of solvent molecules in hydrophobic pockets. However, after about 20 ns, all systems rendered energetically stable. The average RMSD for both APO and complexed PTPRZ was 2.0 Å and 1.6 Å respectively. With the average RMSD less than 2.0 Å, it was deduced that the systems attained conformational stability.^29–31^ The lower RMSD of the NAZ2329-complex relative to the APO system suggest that the inhibitory activity of NAZ2329 possibly induced conformational dynamics that contributed to the system achieving stability at a lower RMSD.

### 3.2 Investigation of the dynamic structural features of PTPRZ WPD-loop

Considering the essential role of the WPD-loop in the catalytic activity of PTPRZ, we explored the atomic flexibility of the residues this loop and the entire protein upon binding NAZ2329. The use of the crystal structures chosen for this particular study was of utmost importance as the binding of NAZ2329 to the allosteric site demonstrated a modification in the crystallized WPD-loop of the protein. To the best of our knowledge, this is the only available crystal structure that demonstrates binding to the allosteric site of the PTPRZ enzyme, thus validating its use for further analysis.

**Fig. 3:**
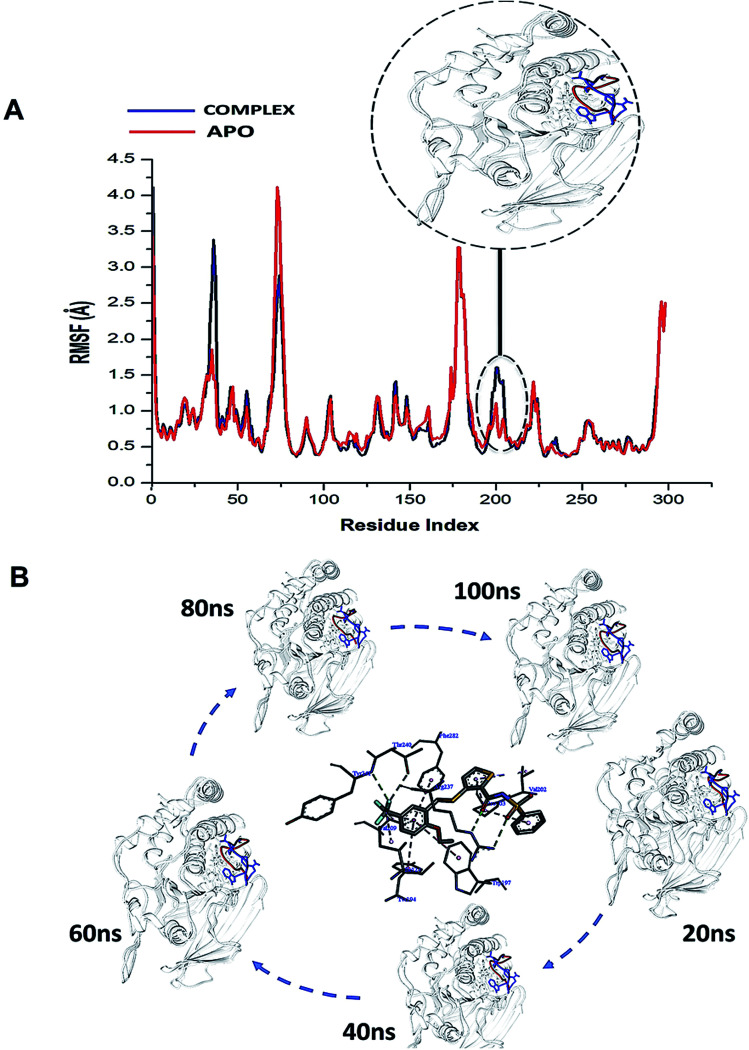
(A) Shows the comparative RMSF plot of the unbound (APO-red) and bound PTPRZ (complexed-blue), (B) Systematic NAZ2329 inhibitory activity in opening WPD-loop across the MD simulation period.

The conformational dynamics of a protein is usually largely dependent on its basic building blocks, thus amino acids,^32^ hence understanding the flexibility of amino acids that make up WPD-loop and PTPRZ could reveal important structural insights on the inhibitory activity of NAZ2329. The ligand induced motions that occur upon the binding and interaction of NAZ2329 to binding site residues, thus triggering a significant conformational change that influence the function of the protein.^32^ As a result of this, we calculated the root mean square fluctuation (RMSF) of the simulated systems illustrated in Fig. 3.

Interestingly, residues 198–204 displayed increased fluctuation in the bound system when compared to that of the APO PTPRZ. This particular region of interest encompasses the WPD-loop, a loop that serves as a flexible gate to the catalytic site of PTPRZ. A “closed” conformation of the WPD-loop denotes an active protein, whilst an “open” conformation indicates inactivity of the protein.^8^ It could therefore be inferred that the allosteric inhibition of NAZ2329 induced an open conformation of WPD-loop, resulting in an inactive protein conformation of PTPRZ. As shown in Fig. 3, the was a consistent higher flexibility of the WPD-loop in the presence of NAZ2329 as the simulation proceeded, suggesting that the allosteric inhibitory activity of NAZ2329 could have induced the increased fluctuation of the WPD-loop in order to ensure its continuous “open” conformation and hence a consequent inactivity of PTPRZ. Overall, the unbound APO and complexed protein appeared to be rigid with the exceptions of select regions, being “175–185”, “71–76” and “34–37”.

**Fig. 4:**
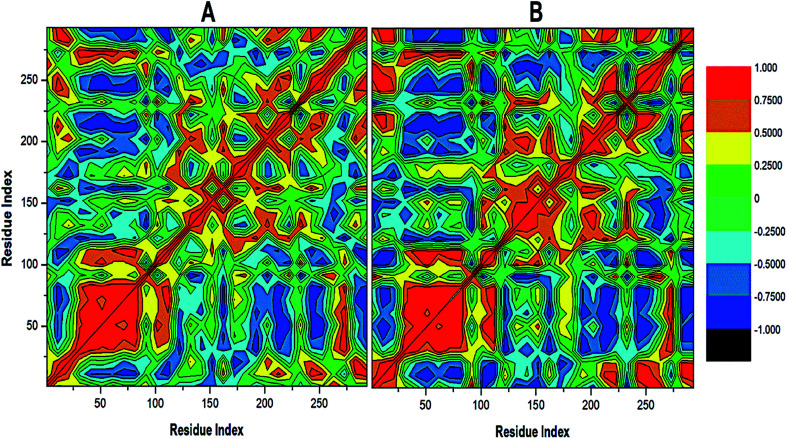
Cross-correlation matrices of the fluctuations of C-a atoms in (A) APO and (B) NAZ2329-complex.

To further reveal the conformational dynamics that occur on PTPRZ due to the inhibitory action of NAZ2329, DCCM matrix analysis was conducted to determine the occurrence of correlated motions over the simulation period based on the positions of Ca atoms of the PTPRZ.^33^ High correlated motion, also referred to as positive correlation, ranges from the colour yellow to deep red (+1), while anti-correlated motions, also referred to as negative correlation, ranges from cyan to black (−1). DCCM analysis revealed that binding of NAZ2329 alters the structure conformation of PTPRZ as shown by the changes in the correlated motions and dynamics. There was an overall anti-correlated motion of residues within the WPD-loop (residues 198–204) in the bound system relative to more correlated motion in the APO system. Since anticorrelated motions of a protein may arise from a significant structural external perturbation such as ligand binding,^34,35^ the observed anticorrelated motion in the bound WPD-loop could be attributed to the allosteric inhibitory prowess of NAZ2329.^36^ This was also consistent with the increased residue fluctuation of the loop in the bound conformation as revealed in RMSF in Fig. 3 as the simulation proceeded. It is also evident from the correlation matrix that a more widely correlated motion is observed in the APO protein, thus confirming conformational shifts after ligand binding (Fig. 4). The residues within regions “71–76” and “175–185” of PTPRZ exhibited anti-correlated motions in both the unbound and bound system which conferred with the observed residue fluctuation results.

**Fig. 5:**
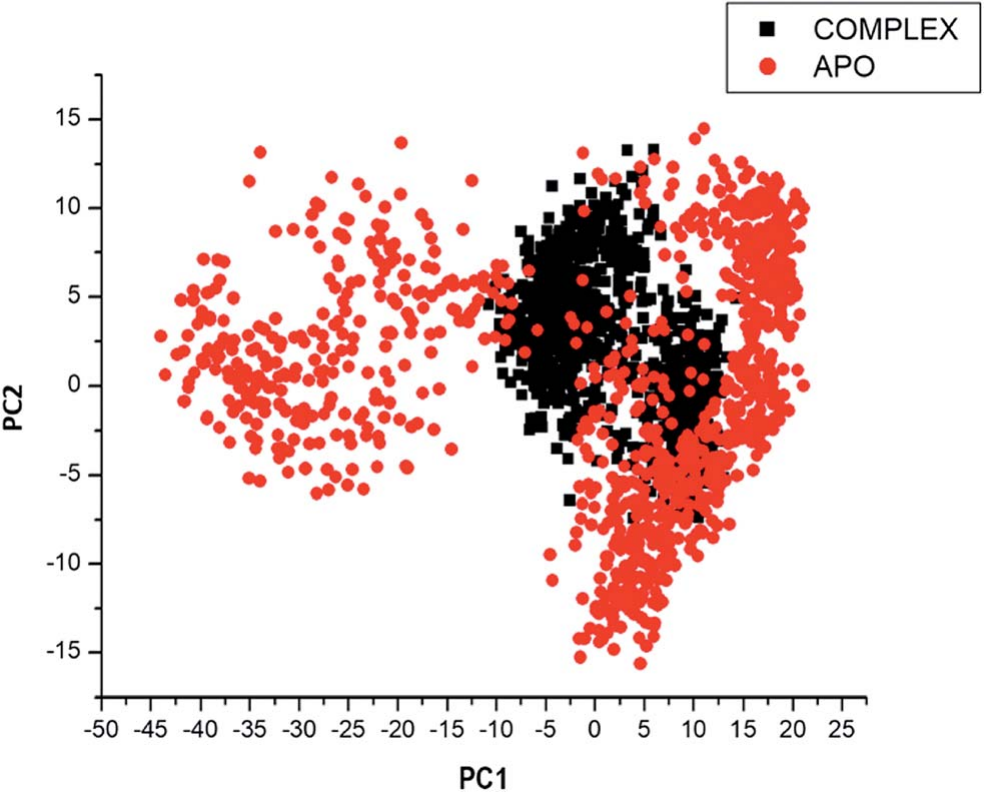
PCA projection of the motion of Ca atoms constructed by plotting the first two principal components (PC1 and PC2) in the conformational space with APO (red) and NAZ2329-complex (black) respectively. PC1 and PC2, respectively, represent a covariance matrix after elimination of eigenvectors (rotational movements). Each point between the single-directional motions represents a unique conformation during the simulation, whereby, similar structural conformations overlap in the graph.

Considering the fact that the biological function of a protein is influenced by its conformational dynamics,^37^ we also employed Principal Component Analysis (PCA), an advanced computational tool to explore the conformational transitions of the APO and complexed PTPRZ over the 100 ns simulation as illustrated in Fig. 4. The clustering method of PCA was employed in this study due to its proven capability of describing varying conformational states generated during an MD simulation. Variations in the conformational states were described by categorizing molecular structures into clusters according to conformational similarities.^38^Fig. 5 highlights the overall motional shifts across two principle components in the case of APO and the NAZ2329-complex.

The simulated systems were projected along the directions of first two principal components (PC1 *vs.* PC2) or eigenvector. The PCA scatterplot generated for the APO and complexed PTPRZ systems shows a significant difference between the two systems along the direction of PC1 and PC2. Overall, the less correlated motion of the APO system confers with the observed higher residue flexibility in the Fig. 4. This implied that the inhibitory activity of NAZ2329 on PTPRZ triggered conformational dynamics as conferred by the conformational flexibility and dynamic cross-correlation analysis. The variation in structural dynamics prompted us to assess the binding mechanism of NAZ2329 to the allosteric site of PTPRZ.

### 3.3 Exploring the drug likeness of NAZ2329

Having been experimental reported to exhibit inhibitory activity against PTPRZ in mouse models, we used online software SwissADME^14^ to determine the physicochemical descriptors, pharmacokinetic features and drug-worthiness of NAZ2329 (Table 1).

**Table 1:** The Swiss ADME Profile of NAZ2329

NAZ2329								
Molecular formula	Molecular weight (g mol^−1^)	Lipophilicity (iLOGP)	Water soluble	GIT absorption	BBB permeability	Bioavailability score	Synthetic accessibility	Druglikeness (Lipinski)
C21H18F3NO4S3	501.56	3.06	Poor	Low	No	0.55	3.77	Yes
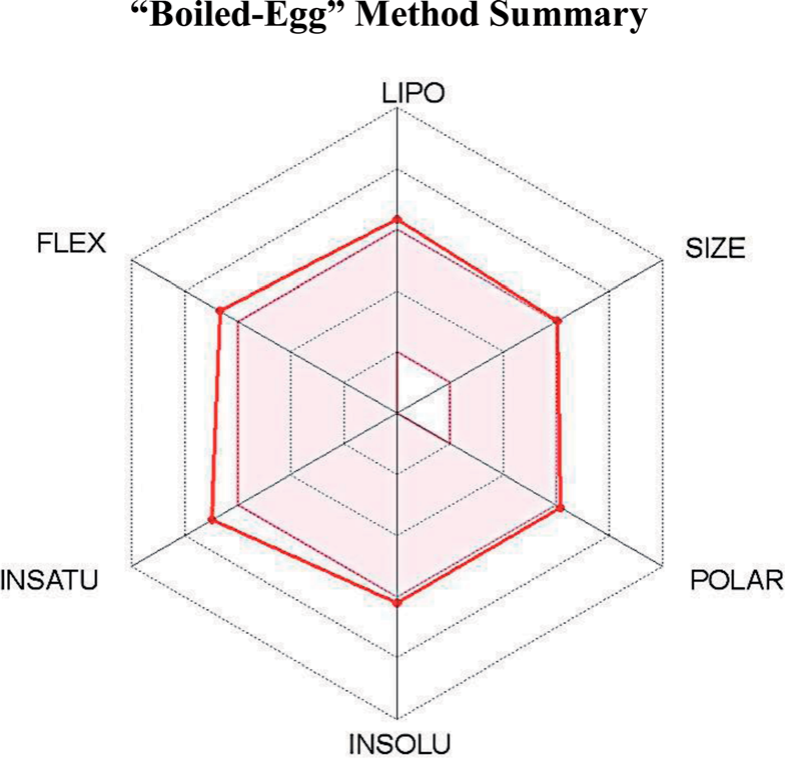								

The lipophilicity and polarity of NAZ2329 was computed using SwissADME, based on the “Brain Or Intestinal Estimated permeation, (BOILED-Egg)” method.^39^ A suitable balance of the pharmacokinetic properties, safety, potency and selectivity is usually paramount in the design of therapeutic chemical agent. Chemical properties such as lipophilicity (log *P*), which are assessed by SwissADME, are very essential features to be considered in the interaction between a chemical compound and its biological target. Usually lipophilicity of a chemical agent influences its permeability, hepatic clearance or solubility. A chemical compound with a log *P* value that ranges from 2 to 3 exhibits a highly favorable potential of achieving permeability and first pass clearance.^40^ However, NAZ2329 showed a log *P* value of 3.06, an indication that NAZ2329 exhibits a less favorable potential of achieving permeability and first pass clearance. Although NAZ2329 exhibited drug-likeness according to Lipinski rules of five,^41^ it has poor water solubility, low gastrointestinal absorption, and cannot permeate through the blood–brain-barrier (BBB). A low gastrointestinal absorption may result in a large amount of the drug being excreted resulting in a possible decrease inhibitory activity. The inability of NAZ2329 to pass the BBB as observed suggests the need for its improvement in its design as a therapeutic agent glioblastoma, a malignancy of the central nervous system.

### 3.4 Estimating the binding mode of NAZ2329 to PTPRZ through free energy calculations

**Fig. 6:**
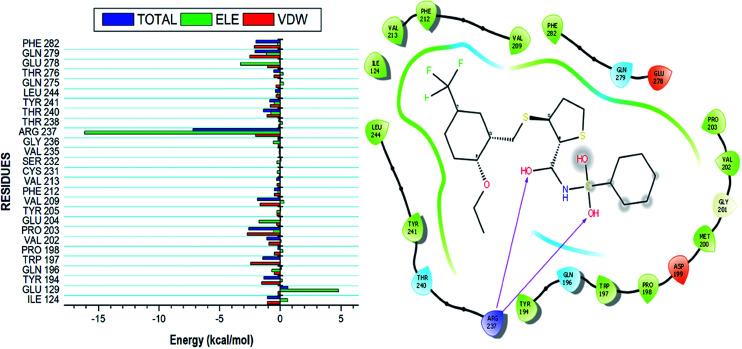
Energy contributions of the interacting residues at the NAZ2329 active site. The residue ligand interaction network illustrates stabilizing hydrophobic interactions pocketing NAZ2329 at the active site. The highest energy contribution was two hydrogen bond interactions shared between ARG 237 with two separate oxygen moieties of NAZ2329.

Using the MM/GBSA based approach; the binding free energy of the NAZ2329-complex was estimated over the 100 ns MD simulation. This revealed the various energy contributions at the catalytic site. To estimate the energies contributed by individual residues located in the catalytic site, per residue decomposition analysis was performed. A depiction of the individual energy contributions and a map of the interactions between the NAZ2329 and active site residues are illustrated in Fig. 6. The estimated relative binding free energy for NAZ2329 is displayed in Table 2.

**Table 2:** MM/GBSA-based binding free energy profile of the simulated NAZ2329 complex^a^

Energy components (kcal mol^−1^)					
	Δ*E*_vdW_	Δ*E*_ele_	Δ*G*_gas_	Δ*G*_solv_	Δ*G*_bind_
Complex	−53.88 ± 0.08	−60.72 ± 0.15	−114.60 ± 0.16	61.98 ± 0.14	−52.63 ± 0.09

a Δ*E*_ele_ = electrostatic energy; Δ*E*_vdW_ = van der Waals energy; Δ*G*_bind_ = calculated total binding free energy; Δ*G*_sol_ = solvation free energy.

NAZ2329 showed a relative binding free energy of −52.63 kcal mol^−1^, implying a favorable thermodynamic stability, which conferred with the compact bound protein upon calculation of radius of gyration as shown in Fig. S2.† Residues that contributed the most to the binding of NAZ2329 include; ARG 237 (−7.201 kcal mol^−1^), PRO 203 (−2.584 kcal mol^−1^) and GLN 279 (−2.064 kcal mol^−1^). From the residue–ligand interaction plot, it could observed, ARG 237 formed the most hydrogen bond interactions hence its high-energy contribution to the total binding energy estimated.

### 3.5 Pharmacophore model creation

**Fig. 7:**
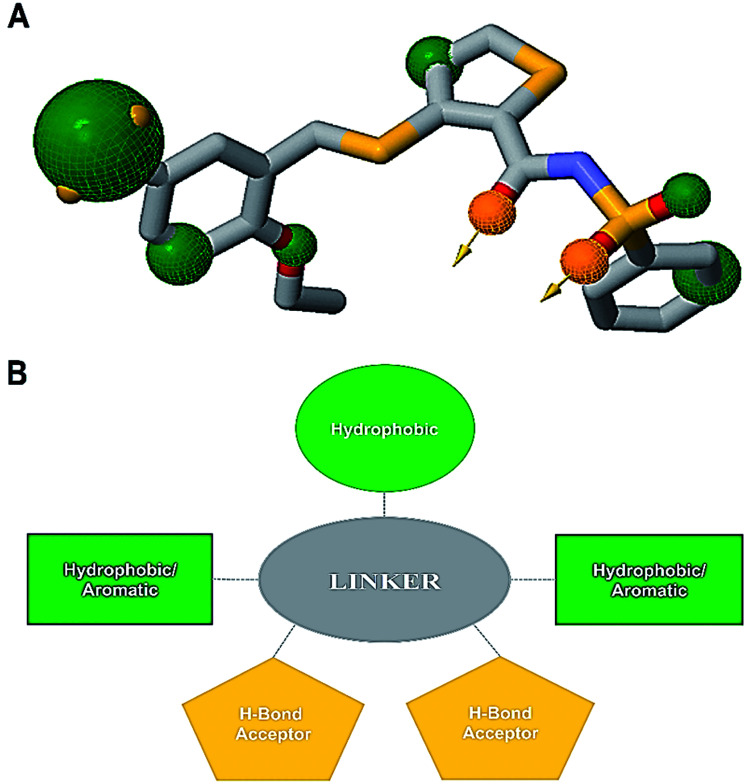
(A) Common pharmacophoric features from alignment of the PTPRZ ligand/residue interaction plots, 3-D pharmacophore model generated on ZINCPharmer (Green – hydrophobic/aromatic moiety, gold – hydrogen bond donor/acceptor). (B) 2-D representation of the chemical features required for potential PTPRZ inhibitors.

An informative structural ensemble of steric and electronic features that were necessary to ensure supramolecular interaction of NAZ2329 with PTPRZ was generated (Fig. 7). This chemical structural scaffold was developed based on the observed NAZ2329–PTPRZ interaction shown in Fig. 6, while taking into consideration the residues that contributed the most to total binding energy as well. The generated pharmacophore model unveils useful chemical insights that could serve as a starting point for the discovery of improved therapeutic agents that target PTPRZ.

## 4 Conclusion

The comprehensive bio-computational analysis employed in this study establishes the structural modifications in PTPRZ subsequent to binding of allosteric inhibitor, NAZ2329. Molecular dynamic simulations divulged profound motional shifts the WPD-loop of the PTPRZ. This flexibility was reported in the RMSF analysis and verified by the graphical investigation of the loop at different time intervals during the simulation. Investigation of the DCC matrix and PCA led to the deduction that the activity of NAZ2329 on PTPRZ triggered conformational dynamics that may be used to explain the mechanism of inhibition of the protein. Based on previous experimental evidence supporting the inhibitory activity of NAZ2329 and the structural dynamics leading to the design of the pharmacophore in this study, we believe that our conclusions could facilitate the design of small molecules that will not only inhibit PTPRZ and PTPRG, but will be applicable to other tyrosine phosphatases as well. This optimized pharmacophoric approach may also lead to a decline in cross-resistance and may increase patient adherence.

## Ethics approval and consent to participate

Not applicable.

## Human and animal rights

No animals/humans were used for studies that are the basis of this research.

## Consent for publication

Not applicable.

## Conflicts of interest

The authors declare no conflict of interest, financial or otherwise.

## Supplementary Material

RA-008-C8RA08427K-s001
