# Supplementary material for: Allosteric inhibition induces an open WPD-loop: a new avenue towards glioblastoma therapy
Source: RSC Adv. 2018 Nov 30;8(70):40187–97. doi: 10.1039/c8ra08427k (PMC9091281; doi:10.1039/c8ra08427k)
Supplement: RA-008-C8RA08427K-s001 [file RA-008-C8RA08427K-s001.pdf]

Electronic Supplementary Material (ESI) for RSC Advances.
This journal is © The Royal Society of Chemistry 2018

Allosteric Inhibition Induces an Open WPD-Loop: A New Avenue Towards Glioblastoma Therapy

Supplementary:

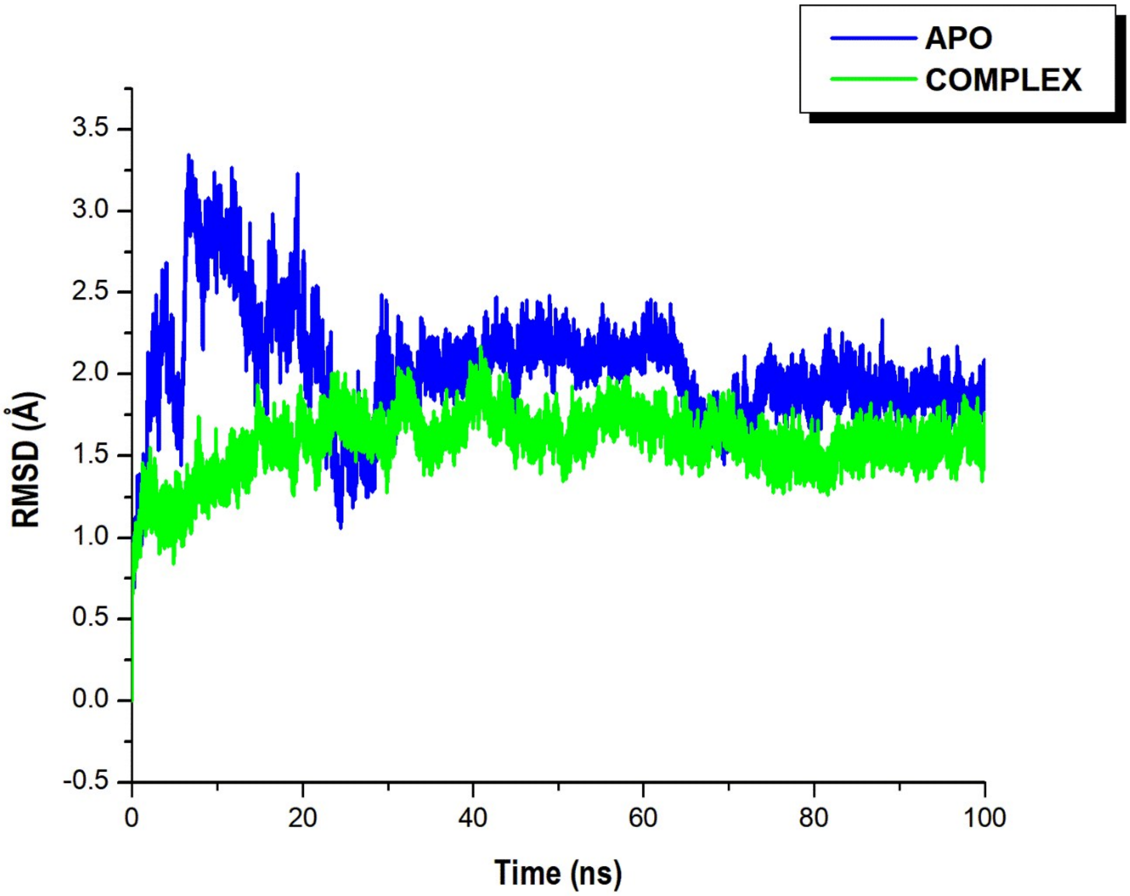

Figure S1: Comparative RMSD of PTPRZ when bound to the allosteric inhibitor NAZ2329 (COMPLEX) and when unbound to any inhibitor (APO).

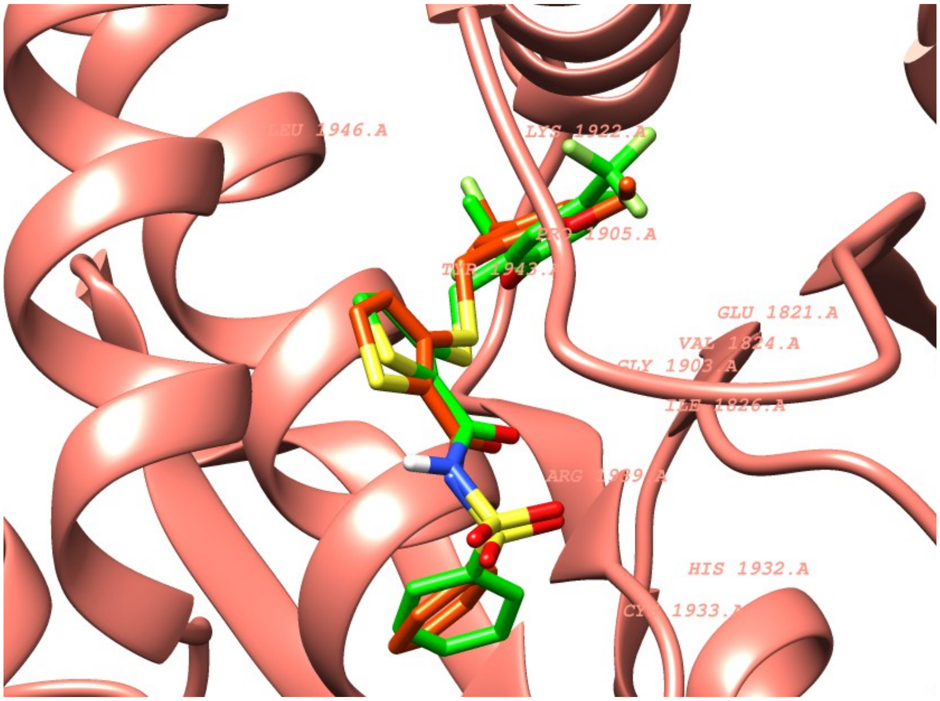

Figure S2: Re-docking of NAZ2329 to allosteric pocket of PTPRZ. Significantly similar docking poses are seen in the docked structure (green) when compared to the crystal structure (orange). This validates the use of the crystal structure for further analysis.

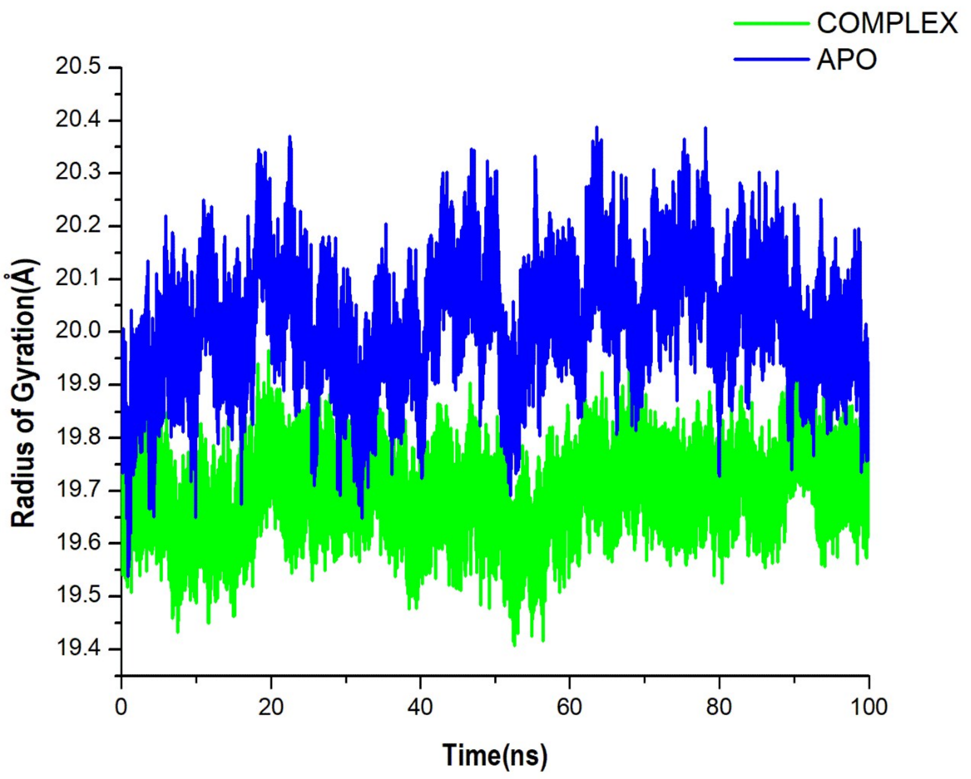

Figure S3: Comparative compactness of PTPRZ when bound to NAZ2329 (COMPLEX) and when unbound to any inhibitor (APO).
